# Factors affecting maternal nutrition and health: A qualitative study in a matrilineal community in Indonesia

**DOI:** 10.1371/journal.pone.0234545

**Published:** 2020-06-16

**Authors:** Sadiq Bhanbhro, Tahira Kamal, Ratno W. Diyo, Nur Indrawaty Lipoeto, Hora Soltani

**Affiliations:** 1 College of Health and Wellbeing and Life Sciences, Sheffield Hallam University, Sheffield, United Kingdom; 2 Department of Community Medicine, Bolan University of Medical and Health Sciences, Quetta, Balochistan, Pakistan; 3 Faculty of Public Health, Andalas University, Padang, West Sumatra, Indonesia; Universidad del Desarrollo, CHILE

## Abstract

The Minangkabau people of West Sumatra in Indonesia are renowned for their matrilineal culture with property and land passing down from mother to daughter. Despite there being a fairly balanced social status for women in the community, the impact of health inequalities is uneven. This study was therefore carried out to explore the relationship between the social, cultural and economic contexts in such a distinctive community with maternal nutrition and pregnancy-related health outcomes, from the perspectives of the mothers, fathers and care providers. Qualitative methods were used to undertake this study in collaboration with partners from the University of Andalas in a suburban area of Padang district. The data collection method was qualitative, semi-structured interviews (n = 19) with women, men, midwives and community health workers. The data were recorded with informed consent, transcribed in the local language and then translated into English prior to being thematically analysed. The major themes which emerged from the data included ‘Minangkabau matrilineality and role of women’; ‘culture and supportive attitude towards pregnant women’; ‘dietary patterns, attitude and access to food’; and ‘limited access to information about food and nutrition’. The findings showed healthy dietary patterns such as regular consumption of vegetables and fruit among the participants. However, the issues of poverty, access to food, dietary taboos and inadequate nutritional information remained major challenges for the mothers and the families who participated in the study. The evidence from this study suggests that the matrilineal culture of the Minangkabau promotes the empowerment of women and offers an encouraging environment for enhancing reproductive health. This lends itself to co-developing locally sensitive and sustainable complex interventions incorporating professional support and building on family and community back-up, enhancing knowledge and demystifying dietary misinformation to improve maternal health and nutrition.

## Background

Tackling maternal health problems and reducing maternal deaths remain major concerns in low- and middle-income countries (LMICs) [1]. The challenging nature of global maternal mortality and morbidity is acknowledged in the United Nation's (UN) Sustainable Development Goals (SDGs) and its precedent document, the Millennium Development Goals (MGDs). SDGs which set a vision to transform our world by 2030 include seventeen major global goals which are related to maternal health, particularly goals 3 ‘Good health and wellbeing’ and 5 ‘Gender equality’ [2].

There are multi-factorial causes of maternal mortality including poverty, lack of access to health-care services and resources, inadequate food and nutrition, and health-seeking behaviours and belief systems [3]. Pregnancy is a distinct phase in the life of a mother providing an opportunity for applied interventions to reduce health inequalities for mothers and their families. The nutrition of mothers during pregnancy plays a vital role in the short- and long-term health of a mother and her growing foetus [4]. However, maternal nutrition is usually subject to contexts within the society in which a woman lives. These include social, cultural, economic, political, environmental and behavioural systems which impact on the nutritional wellbeing of pregnant women. For example, it has been suggested that cultural belief, particular taboos and attitudes of women, their partners and their families influence their choices about what to eat and what not to eat during pregnancy [5].

Understanding the relationship between maternal nutrition and health through the lens of wider social determinants requires a deep exploration of how different factors, processes, power constellations, institutions and interests affect the maternal nutrition and health within household, community, culture and region. Gender disparities in access to and control of resources might curtail women's potential productivity and increase their health risks, and these have therefore been the focus of investigations in several countries [6]. Maternal nutrition practices, access to resources and food distribution systems which are ultimately impacted by socio-economic, cultural, family and community settings might determine reproductive health, particularly for vulnerable groups, making them more susceptible to conditions such as anaemia, malnutrition, malaria and other illnesses during pregnancy. The relationship and the impact of health and nutrition during pregnancy could be specific to particular situations related to the social status of women. Therefore, communities with a unique social organisation and gender status such as that in the case of the matrilineality of the Minangkabau provide special conditions for exploring some of pertinent concerns related to health, nutrition and social settings.

The Minangkabau (also known as Minang, hereafter Minang) are an indigenous ethnic group in Indonesia renowned for their long-held matrilineal tradition, or *matriarchaat* (from the Dutch), which is today cherished equally by their women, men, boys and girls. The Minang *matriarchaat* is an established social system which appears to be drawn largely from *adat*, another old Minang tradition which involves tracing inheritance through the matrilineal line and gives prominent roles to women in public ceremonies. Minang women uphold these customs, which not only trace ancestry through the female line but also involve a complex social system in which women and men share power and control based on the principle of inter-dependence and mutual responsibility [7]. This community is different from the rest of Indonesia’s ethnic groups in many ways, but the most significant difference is their unique matrilineal family system, in which gender is a major factor for inheritance. The ownership of property (such as land, house or livestock), for instance, must pass from mother to daughter, whereas in other ethnic communities of Indonesia, inherited property passes from father to son, which is the common practice among Muslims and other religious groups around the world. So, the reckoning of inheritance and descent through the female line seem to be empowering for Minang women to enjoy a fairly balanced status, but the impact of health inequalities is uneven, with clear evidence of poor maternal health outcomes in these communities [8]. The social status of Minang women is considered empowering in the sense that it imparts power to the women to negotiate and take an active part in day-to-day decision-making [9].

Indonesia is the world's fourth most populated country, administratively divided into 34 provinces, 98 municipalities and 410 districts [10]. The country has around 13,000 islands with hundreds of diverse ethnic groups [11]. Health-related indicators in Indonesia have improved, with life expectancy rising from 63 years in 1990 to 71 years in 2012, under-five mortality falling from 52 to 31 deaths per 1,000 live births and infant mortality falling from 41 to 26 deaths per 1,000 live births between 2000 and 2012 [12]. However, the maternal mortality ratio (MMR) has remained high with 210 deaths per 100,000 births in 2010, and stark inequalities persist across regions. For example, West Sumatra (the home of the Minang people), is ranked thirteenth out of 33 provinces with an MMR of 212 per 100,000 live births [8].

There have been several studies which have investigated the association between economic factors and this situation: local food consumption patterns, food supplementation interventions, women empowerment strategies and maternal nutritional status and health [13,14,15,16]. However, the literature on relationships between health, nutrition and matrilineality as a unique social organisation which places women in a distinctive position within the society, is limited. This qualitative study, informed by a social determinants of health model which addresses the socio-economic, cultural, environmental and political “conditions in which people are born, grow, live, work and age” [17] was therefore designed to provide an in-depth understanding of the relationships between the social, cultural and economic contexts in such a unique community, with maternal nutrition and pregnancy-related health outcomes.

### Aim and objective

The aim of the study was to identify the possibilities for developing culturally appropriate interventions to improve maternal health and nutrition in a widely recognised matrilineal community of Indonesia. In order to address this aim, the principal objective was to explore factors which affect the nutritional well-being of pregnant women in relation to women's cultural status as well as access to and the availability of food from the perspectives of women, their partners and health-care professionals.

### Key messages: (3–5 messages of 80–100 words)

1. The Minang matrilineal culture promotes empowering women who have real power, such as taking part in decision-making, leading community ceremonies and having ownership of resources–land, water and rice paddies. It therefore offers an encouraging environment in which to develop locally sensitive and sustainable interventions to improve maternal health and nutrition.
2. Some pertinent food taboos and cultural beliefs were identified which might have inhibited a healthy dietary pattern with potential adverse outcomes for pregnancy.
3. The study highlighted avenues for potential interventions to address issues such as food taboos and improving access to adequate information related to nutrition.

## Methods

This section describes the theoretical underpinnings which informed the study, the research setting, the participants, the data collection tools, the data analysis and the obtaining of ethical approval. It should be noted that the data in this paper are reported in accordance with the Standards for Reporting Qualitative Research (SRQR) using the consolidated criteria for reporting qualitative studies (COREQ), which is a 32-item checklist [18].

### Theoretical framework

Considering the exploratory nature and objective of this study, the quantitative research methods such as laboratory-based trials, polls, questionnaires and surveys were deemed unsuitable. In contrast, the qualitative interviewing methods based on the social constructionist paradigm that there are multiple, local and specific ‘constructed’ realities was used to elicit the participants’ perspectives on the research topic. The critical medical anthropological theory [19] in conjunction with a social determinants of health model underpin the study. The methodological and theoretical orientations were used to explore and understand how the matrilineal cultural context influences and shapes the nutritional health of mothers in the Minang community and to identify possibilities for addressing maternal nutritional health issues. Given the lack of previous research on these aspects of the community, the data collection methods needed to have the potential to engage potential participants and encourage them to provide their perspectives on the issue being studied. In view of the exploratory objective of the study, semi-structured interview was therefore used as the data-collection method.

### Setting

The study was carried out in a block of a suburban area of Padang district in West Sumatra province. The province lies on the west coast of the island of Sumatra and had an estimated population of over five million people in 2014. West Sumatra is sub-divided into twelve regencies and seven cities. It has relatively more cities than other provinces in Indonesia, except for Java province. Padang is the largest and capital city of West Sumatra province and has a population of over a million. The Minang community constitute the great majority of the population. The community is renowned for its long-held matrilineal tradition and known for its people’s devotion to Islam. Rice, fish, coconut, vegetables and chili are considered the main food items consumed every day in a Minang household. The best-known cuisine in Minang food culture is *rendang*, a spicy beef stew [20].

### Participants

The study participants were from the Minang ethnic group and spoke both Minang and Indonesian *bhasha* (language). Semi-structured interviews were conducted with pregnant women and/or women who had been pregnant in the last twelve months (n = 7), their husbands (n = 3), mothers and mothers-in-law (n = 4), health-care professionals (n = 3) and *Kaders*—volunteer community workers (n = 2). A purposive sampling strategy was adopted using the following inclusion criteria: participants were from the Minang community due to their unique social organisation of a matrilineal family structure; pregnant woman or pregnant within the last twelve months; partner of a pregnant woman; mother or mother-in-law of a pregnant woman; and health-care professionals working with pregnant women, such as midwives. The participants were approached by phone and face-to-face through the health-care professionals and a *Kader*. In total, twenty participants were approached, of whom nineteen were interviewed; the one who declined to be interviewed had to go out of town in an emergency. Prior to the interviews, the participants were informed that one of the researchers was from a UK university and that the local researchers worked in Unand (Andalas University). Additionally, the local researchers did not receive any extra money for working on this project except for their research expenses, such as transport cost, meals and drinks, which were paid from the project budget. Apart from the interviews, the study participants had no opportunity to comment on or offer corrections to the transcripts or the findings.

### Data collection

Qualitative semi-structured individual interviews were used to collect data for this study. The interviews were conducted by a male (RW) and two female (WI and JF) local researchers from the Indonesian partner institution. All the researchers were university graduates and had experience of qualitative interviewing. None of the researchers involved in the study had any relationship with the study participants. In addition to the local researchers and the participants, the first author (SB), a male researcher from the UK, was also present during the interviews. This researcher was trained in anthropology and public health and his key role was to make observation notes during the interviews. It appears from the data and the debriefing meetings with the interviewers that his presence had no significant influence on the data collection as the researcher did not know the local language and was not aware of the cultural subtleties of the population, so his presence was seen as a ‘silent guest’. Moreover, according to the local researchers, the UK researcher looked like them (the local population) as he was from an Asian ethnic background (observation notes). The influence of the local researchers on the research processes could not be assessed as they did not maintain field diaries or document their observations.

All the interviews were conducted face-to-face and took place in the Community Health Care Centre and participants’ homes; each interview lasted between 45 minutes and an hour. The interviews were audio-recorded after obtaining informed consent, then translated and transcribed by the researchers.

### Ethical approval

Ethical approval for the study was obtained from the relevant institutions’ ethical approval committees. Full information was provided for eligible participants prior to the study commencement and they were assured that their data would be anonymised with full respect given to confidentiality. It was made clear to the participants that their participation was voluntary, that their decision to take part in the study would not affect their routine care and that they were free to withdraw from the study at any point during the process.

A generic interview guide was developed in collaboration with the Indonesian research partners prior to the fieldwork in order to ensure that appropriate questions and prompts were used (*see* the interview schedule in S1 Appendix). Given the different characteristics of the participants, variations in the interview guide questions were made during the interviews; for instance, some of the questions were specifically put to the pregnant women and those were modified for their partners or family members. A similar principle applied for the interviews with the health-care professionals.

One of the local research assistants translated the interview guide into the local language and another senior staff member from the faculty verified the translation. The interview guide was not rigid or prescriptive but was a checklist to ensure that all the topics were covered which were intended to be explored in the study [21].

### Data analysis

The inductive or 'bottom-up' approach [22,23] of thematic analysis was used to analyse the data manually. Theme-led data coding was used in which the pertinent themes were identified from the data without trying to fit them into a predetermined coding or theoretical framework [24]. The local researchers (RW & WI) translated and transcribed the interviews into English. The translation and transcription of the interviews from Indonesian *bhasha* into English was double-checked by two senior staff members from the faculty. The data transcripts were coded by the two researchers (SB & RW) and verified by the third reviewer (HS) to enhance validity. The analysis comprised the following five steps: 1) reading and re-reading each item of the data set; 2) producing initial codes from the data; 3) identifying themes and then sorting the codes into the identified themes; 4) defining and further refining the identified themes; and 5) writing the narrative of the analysis.

## Findings

### Characteristics of the participants

Fifteen women and four men, including health-care professionals, took part in the study. Most of them were originally from Padang city and a few had migrated from other parts of West Sumatra province; *see* Table 1.

**Table 1:** Demographic information of the study participants.

Participant no.	Sex	Age	Education level	Pregnant/had pregnancy in the last 12 months	No. of Children	Occupation
1	F	46	Bachelor’s degree	No	3	Health-care professional (midwife)
2	F	34	Bachelor’s degree	No	1	Health-care professional (midwife)
3	F	42	Junior High School	No	2	*Kader* (voluntary community worker)
4	F	30	Senior High School	Yes	0	Homemaker
5	F	22	Bachelor’s degree	Yes	0	Homemaker
6	F	58	Senior High School	No	3	Homemaker
7	F	30	Diploma	Yes	1	Teacher
8	F	26	Junior High School	Yes	0	Homemaker
9	F	27	Senior High School	Yes	0	Homemaker
10	F	48	Senior High School	No	2	Homemaker
11	F	18	Junior High School	Yes	0	Homemaker
12	F	24	Bachelor’s degree	Yes	0	Homemaker
13	F	37	Senior High School	No	3	Homemaker
14	F	40	Elementary	No	4	*Kader* (voluntary community worker)
15	F	31	Elementary	Yes	2	Homemaker
16	M	25	Junior High School	NA	0	Motorbike taxi (*ojek*) driver
17	M	36	Bachelor’s degree	NA	1	Health-care professional (In charge of the health centre)
18	M	38	Bachelor’s degree	NA	2	Self-employed
19	M	26	Elementary	NA	0	Shopkeeper

All (n = 19) participants in the study owned a mobile phone and half of them had access to the internet. Most of the households owned at least one motorbike, which is the main mode of transportation there. Six participants had their own house and all the others were renting from local landlords.

### Identified themes

The major themes which emerged from the data included: 1) Minang matrilineality and the role of women; 2) culture and supportive attitude towards pregnant women; 3) dietary patterns, attitude and access to food; and 4) limited access to information about food and nutrition.

### Minangkabau matrilineality and the role of women

The participants discussed various characteristics of the Minang people but reported four key characteristics, matrilineality, devotion to Islam, out-migration and Minang cuisine, which make their culture distinctive. Matrilineality is an established social system that involves tracing inheritance and property such as houses and land through the female line, giving prominent roles to women in public ceremonies and providing them space to share power with men based on the principle of inter-dependence and mutual responsibility. In day-to-day activities, the role of Minang women may seem traditional, such as house-hold chores and looking after children and elderly relatives, but their sense of equality with men and shared power might not always be consistent. The women actively contribute to the economy; they are farmers, entrepreneurs, industry workers and teachers, but there can be differences in their economic status depending on whether they are from urban or rural areas. For example,

In villages, the economic position of women is strong compared to women living in small towns or sub-urban areas because the major part of food production such as rice and beans is owned by them. (male participant 18)

The ownership of food products gives women an advantage in decision-making especially in the areas which are in their control. Minang women play crucial roles in all spheres of life from the household to the market. A closer look at various aspects of Minang customs and traditions showed that women exercise power in a real sense. The same participant stated,

From grocery shopping to kids’ education, my wife has the final say. This doesn’t happen only in our house but almost in all Minang families, similar standards are followed. (male participant 18)

Women have a central role in community ceremonies and ownership of resources–land, water and rice paddies–and these roles and responsibilities give them special status in the community. One of the male participants said,

The core values and traditions of Minang culture are female oriented; hence there is no preference for boys over girls, also no differences in education and health-care for boys and girls. All men and women are treated equally. You can see girls driving motorbikes. You can't see such things in many Muslim countries. (male participant 17)

The ceremonies led by women are weddings (*baralek*), harvesting (*manyabik*) clan leader inaugurations (*batagak pangulu*) and many more. This shows that women are at the centre of Minang culture as these are not only displays of influence and the contribution of women to social activities, but the leading role of women in these ceremonies plays an important part in passing these traditions and cultural roots on to the next generation. Women as mothers are cherished by the community, as their status not only provides ownership of the family property but the major responsibility of the upbringing and socialisation of the children. For instance, a participant stated that,

In the community, girls are preferred children and their socialisation is the primary responsibility of a mother because she is passing the role on to the future mothers. (female participant 11)

The participants were of the view that the changes in state policies and economic pressures may in some instances have increased men’s claim to power and authority in the Minang community, especially in the current political sphere. However, women enjoy the upper hand in day-to-day decision-making involving household management: they decide on the budgeting, shopping and children’s education. This was described by several participants:

I can buy whatever I like from the market for eating, drinking and wearing–my husband can’t interfere in it. (female participant 7)

My wife doesn't have land or any other property, but she enjoys the same status in my household and in the community as other women who do own property; she is the boss of our house because of our tradition. (male participant 16)

Strains do happen between wife and husband because of other pressures such as inflation, unemployment, decline in family income but we always try to resolve them amicably, this is because of our long-held tradition of *adat*. (female participant 9)

The centrality of women in traditional ceremonies and matrilineal customs not only empowers them to lead and contribute to the social and cultural activities but also provides opportunities for them to receive proper attention and support when they need it, particularly during vulnerable periods such as pregnancy.

### Culture and supportive attitude towards pregnant women

There seems to be a strong sense of cultural support and a positive attitude towards pregnant women both from the family and within the community. One of the key features of Minang culture is that when couples marry, the groom moves to the bride’s house and lives with his in-laws. This makes a woman's position special as she will continue living with her parents and when a woman becomes pregnant, not only her husband but also her parents will be looking after her. For instance, participants expressed their views as,

I enjoy my pregnancy time because there are many people including my husband, mother, father and a younger sister, they all look after me. In addition, regular visits from my husband’s family too. (female participant 6)

My delivery days are close, so I need someone with me all the time; when my husband goes to work, my mother takes care of me. (first-time pregnant mother 4)

Staying at her parents’ house, especially during pregnancy, is not only comforting but a kind of luxury which we Minang women have because of our culture. (volunteer community worker 14)

In some situations, after a wedding, staying in your parents’ house has disadvantages too, particularly, when your parents are old and your husband has gone abroad for work and his family lives far from you; in such a situation managing pregnancy is difficult. (female participant 5).

A healthy diet and nutrition throughout pregnancy have a significant impact on the health and wellbeing of a mother and a child. All of the women participants, including midwives and *Kadres*, stated that they do get whatever they want to eat and drink but that this depends on the financial position of their household. They were also of the view that it is women’s responsibility to prepare food for the family, but that during pregnancy they are relieved from not only cooking but also other household tasks such as cleaning, washing and making beds. One participant said that,

Sometimes I am tired of just eating, sitting or staying in bed. It is good to be looked after, but sometimes it makes me sick. (mother with a new-born 6)

In contrast, another participant stated that,

I enjoy being pampered, pregnancy is a special time in our lives, therefore we deserve special treatment too. (pregnant mother 10)

All of the participants stated that the special status of women in Minang culture has many advantages for women in pregnancy and in other aspects of their lives, but there are many other socio-economic and political factors which have an immense impact on the health and wellbeing of pregnant mothers, such as the financial condition of their family, the knowledge, attitude and behaviour of women and other family members towards food and nutrition, access to food products, and government policies and the socio-economic context within the communities.

### Dietary patterns, beliefs and access to food

Rice, fish and coconut are considered the key constituents of Minang food, along with *rendang* (a popular meat dish). Vegetables and fruits are consumed regularly. Chili, ginger and turmeric are the most commonly used spices and herbs in Minang cooking. The consumption of rice is more frequent than other starchy food items. For instance, one of the respondents recounted that,

We eat rice four times a day during pregnancy, which is more than usual consumption. (female participant 5)

It was observed that despite the availability of a range of starchy food varieties such as corn, cassava and yams, only a few types of food dominated the overall dietary pattern, with rice being mainly used, or in some cases wheat, within these communities. Among the Minang, rice is the most important food as one male respondent explained,

If I don’t eat rice, that means I haven’t had my dinner and I won’t be able to sleep without having rice. (male participant 18)

Eating meals three times a day is a common pattern: breakfast, lunch and dinner. However, the interviewees mentioned that this has been changing due to an emerging new lifestyle:

In urban areas, missing breakfast is more common than in rural villages and towns where people still don’t go out in the morning without eating breakfast. (health-care professional 17)

A midwife added that,

In cities regular snacking a few times a day is trendy rather than having proper meals; this is becoming the trend in pregnant women too and could be harmful for those who don’t know about the differences between nutritious and oily and sugary foods. (health-care professional 1)

This was reinforced by the second midwife participant:

In particular, the increased availability of fast food, including noodles, potato chips and fried chicken, have made the situation more problematic as people prefer to buy food from fast-food chains for their convenience rather than cooking a balanced diet at home. (health-care professional 2)

Like the matrilineal tradition, Minang people are proud of their cuisine too. A couple of the respondents proudly said that their “food is very popular, delicious and spicy; one can find Minang restaurants in all parts of Indonesia and abroad too” (female participants). Another participant went on to explain that "even the then American president Obama liked our food, *rendang*, it was presented to him when he was visiting Indonesia" (male participant 19).

The participants stated a wide range of beliefs about what types of food should be avoided during pregnancy; views about hot and cold foods were particularly persistent. Largely, a key belief was that all food items which are hot in nature should not be consumed because they can cause many difficulties during pregnancy such as miscarriage, premature delivery, bleeding and sickness. Some of the respondents described this:

Young coconut water and fermented cassava should not be consumed during pregnancy because both are hot, so they might cause a miscarriage. (volunteer community worker 3)

Instant noodles, radish, ice, coconut, fermented cassava–all these foods are avoided in pregnancy because they can cause complexities, such as ice and radish will cause the baby to bloat and fermented cassava is a hot meal so it could cause a miscarriage. (female participant 7).

It is important to note that most of the participants were of the view that vegetables are better than meat. One-woman participant said that,

Eating meatballs is not good in pregnancy because they contain additives, which could cause problems. (female participant 4)

Another woman respondent believed that,

Consuming meat frequently will make breast milk bitter, therefore it should be avoided. (female participant 9)

Interestingly, only one-woman participant was against eating durian fruit in pregnancy because of its strong odour; others were not clear about its perceived impacts. When women were asked about how they knew about the hot or cold nature of food, they were of the view that this information has come down through the generations. A couple of the participants said that they knew from their personal experience and learnt from the experiences of their friends and neighbours too (female participants). These attitudes of pregnant women about the benefits of consuming vegetables and fruits are valuable but their perceived disadvantages of eating meat could put them at risk of iron deficiency and anaemia [12].

In addition to their dietary patterns and attitudes towards food consumption, the accessibility and affordability of food were important issues emerging from the data. The participants discussed the rise in food and fuel prices and the low income of their households as factors affecting the nutrition and food consumption of pregnant mothers. They expressed their concerns that there was not much state support for mothers except for the provision of vitamin supplements to pregnant women for a limited time. Some of the pregnant women participants commented that their household income was very low, and they could not even afford the cost of daily living. Although pregnant women face increases in the cost of food and medicine, there is no additional support from the authorities to ease their financial challenges. The participants showed their concern about this:

Midwives and doctors advise us to eat and drink a variety of things like apples, pineapples and soya milk during pregnancy, but many of us can't afford these things. (female participant 12)

On the same lines, a *Kader* said that,

Typically, women eat three times a day but during pregnancy they need to eat four to five times a day, plus snacks two or three times a day; that means they should buy more food items but again the issue is money. Those who have money can eat a healthy diet, but the problem is for the women who can’t afford it. (voluntary community worker 14)

This shows that affordability is a real concern for people because it has a severe impact on the nutritional wellbeing of pregnant women, particularly in households with a low family income. However, except for these financial issues, other challenges regarding access to food such as transport, the non-availability of local grocery shops or the quality of food were not discussed.

### Limited access to information about food and nutrition

Another important theme which emerged from the data was the inadequate access to information about food and nutrition. All of the women participants stated that they received information during pregnancy from a variety of sources such as parents, in-laws, midwives, a *Kadre*, television, radio and traditional birth attendants. Several participants valued the guidance and information on all aspects of pre- and post-pregnancy from their mothers and mothers-in-law along with *Kaders*. One participant said,

I prefer to follow my mother's suggestions during a pregnancy because she has experience and good knowledge of pregnancy and childbirth. (female participant 3)

Many stated that they preferred to receive relevant information from health-care professionals because they thought that it is more reliable. Their views about the reliability of advice and about the professional hierarchy varied, as some expressed their positive views about midwives and *Kadres*, saying that they are good sources of information:

I think that regular counselling from midwives during pregnancy is the best source of information for a normal pregnancy, delivery and beyond. (female participant 9).

However, one participant thought that doctors are the best because she believed that "doctors have more knowledge than other health-care professionals*"* (female participant 6).

With regard to the route of communication and means of receiving information, many of the participants preferred face-to-face exchange of information, but the younger participants commented that they would like to get information through their mobile phones and through text messages.

## Discussion

This exploratory study has highlighted several facilitating factors such the Minang matrilineal culture and supportive attitude towards pregnant women within the family and the community. There are, however, alarming issues related to dietary patterns and to attitude and access to food which are important to consider when planning interventions to tackle health inequalities in these populations. Challenges identified such as poverty, economic restrictions, lack of access to adequate sources and insufficient information are somewhat modifiable and given the social status of women in these communities being of a favourable nature, interventions directed at women as a focal point for families would be a good start. Previous studies of pregnancy have paid little attention to investigating inter-relationships between social organisations and the status of women in a community or the nutritional wellbeing of women during pregnancy in Indonesia. For this study, we used qualitative methods to explore and understand the relationship between the unique Minang matrilineal culture and the nutritional attitude and behaviour of pregnant mothers. The participants believed that Minang matrilineality empowers and allows women to make decisions during pregnancy about nutrition and the management of the household resources; however, major factors such as low income, no support from the government, limited access to nutritional information, and food-related cultural beliefs have a negative impact on their pregnancy. The findings suggest that matrilineal customs, traditions and practices have a positive impact on families' outlooks towards health and supporting women, particularly during pregnancy. The participants were appreciative of receiving appropriate support and attention from family members and they wanted to receive robust information during pregnancy for a healthier outcome. The women particularly enjoyed their independence and special status within the household and the community due to the matrilineal Minang culture, with the freedom to eat food of their choice. However, other challenges such as taboos about particular foods and socio-economic constraints may have inhibited a healthy dietary pattern with potential adverse outcomes for pregnancy. The participants believed that there are some foods which should be avoided during pregnancy and some types of food which should be consumed in pregnancy. Their stated food taboos expressed preferences for vegetables over meat; however, meat as a good source of iron and B12 vitamins is important to be consumed unless careful alternatives are included in the diet to prevent anaemia [25]. In a previous cohort study, we observed a high rate of anaemia (45% in the 2^nd^ trimester of pregnancy) in Indonesian women [13]. Where anaemia is a challenging burden, particularly during pregnancy with its known adverse outcomes such low birthweight and preterm birth, demystifying such traditional myths through appropriate information-sharing pathways is essential [26,27]. The findings regarding food taboos and avoiding essential nutrients are in line with those of other studies which have shown beliefs about hot and cold food which are widely observed in many cultures around the world, such as China, India, Malaysia, Mexico and other parts of Indonesia [28,29,30,31,32,33]. There is a cross-cultural variation in knowledge, beliefs, origin and purposes related to such food taboos [34]. Understanding these dietary restrictions in communities with a high level of maternal mortality and known nutritional deficiencies could be helpful for finding appropriate strategies to enhance nutritional knowledge and reduce the risk of related adverse outcomes for mothers and babies.

Using the social determinants model, it was discerned from the data that the household income is the key factor in the nutritional wellbeing of women. Although the Minang matrilineal culture empowers them to make choices, family income and poverty inhibit them from an adequate access to the correct nutrition. For instance, if food is expensive, households with a low income have to buy and eat cheap and less nutritious foods, which has negative consequences on pregnant women leading to or exacerbating conditions such as anaemia and under-nutrition in these vulnerable populations.

The pregnant women participants considered health-care professionals, and particularly midwives, as a credible source of information which conforms with other studies' findings [35,36,37]. These studies have demonstrated that midwives are perceived as reliable sources of information because of their close contact with pregnant women and an appropriate training and education. The participants also trusted and relied heavily on the information which was provided by their mothers and family members due to their personal experience and knowledge. However, concerns were raised about the lack of sufficient information for mothers and their families regarding nutrition and diet for optimising pregnancy outcomes. Our findings show that despite a positive socio-cultural attitude towards women’s status, concerns over a lack of varied healthy nutritional and dietary patterns due to food taboos, economic constraints and the lack of sufficient information persist. These findings provide an important evidence base which could inform the co-production of appropriate interventions, building on a supportive community and family infrastructure, to enhance dietary knowledge and behaviour during pregnancy with the ultimate aim of improving maternal and infant health and wellbeing.

### Strengths and limitations of the study

This is one of the few (if any) studies which have focused on providing a better understanding of the relationship between cultural context and gendered social positioning with the nutritional health and wellbeing of pregnant mothers. A key strength is the collaborative nature of the work, which involved working closely with local researchers in all aspects of data collection and interpretation of the findings. The study also included a wide range of stakeholders including not only women (pregnant or new mothers) but also their partners, mothers and in-laws as well as health providers to allow a much richer state of understanding.

The small number of participants could be a limitation of this study, however for an in-depth explorative study particularly on an international level this could be a good start to begin demystifying socio-cultural and nutritional beliefs in relation to pregnancy health and wellbeing. The language barrier was another limitation of such an international study. However, in close collaboration with the local researchers, every care was taken in interpreting the interview transcripts with cross-validation by other research team members to reduce the risk of bias.

## Conclusion

Despite the fairly balanced social status of Minang women due to the matrilineal traditions in their households and the community, the impact of health inequalities on women’s health remains a challenge. Issues such as poverty, access to food and adequate/appropriate nutritional information during pregnancy seem to be the major challenges for pregnant mothers and their families. The study has demonstrated that the Minang matrilineal culture promotes empowering women and offers an encouraging environment and an opportunity to co-produce and evaluate locally sensitive and sustainable interventions to improve maternal health and nutrition.

## Supporting information

S1 Appendix Interview guide. (DOCX)
