# Supplementary material for: Factors affecting maternal nutrition and health: A qualitative study in a matrilineal community in Indonesia
Source: PLoS One. 2020 Jun 16;15(6):e0234545. doi: 10.1371/journal.pone.0234545 (PMC7297355; doi:10.1371/journal.pone.0234545)
Supplement: S1 Appendix — (DOCX) [file pone.0234545.s001.docx]

**Supporting Information**

**Appendix 1**

**Project: Factors affecting maternal nutrition and health: A qualitative study in a matrilineal community in Indonesia**

**Interview Guide**

Date (day/month/year): ________Time interview began: __________

Name of facilitator: ___________Time interview ended: __________

Name of the interviewer: ___________________

**Introduction**

Today we will be discussing some issues that are important to women, men and children in your community that is nutrition and health, including social, cultural, economic and behavioural factors and their impact on nutritional wellbeing of pregnant women. We are interested in learning about opinions and practices of people in your community including your own, as well as how information about maternal nutrition is learned.

1. **Would you describe your family structure?**

**[Probe]:**

- Is it joint/extended/nuclear family?
- How many people live in your house?
- What is the major source of income in your household?
- Where do you get groceries or food products?
- Who does get groceries of food products in your household?

1. **Would you describe do women in your community have access to productive resources such land, water, labour and credit?**
2. **Would you describe, who do you think is healthy and having a good pregnancy and who is sick or might be having problems with her pregnancy.**

[Probes]:

- Why are the women either healthy or sickly/with problems?
- Is their diet an influence and if so what is it about their diet?
- What is the experience of women in this community with delivery/birthing?
- Is the experience connected to her diet during pregnancy?

1. **How aware are people in your community about maternal nutrition?**

[probe]:

- Do your neighbours or friends know what maternal nutrition is?
- What do people in your community think maternal nutrition is?
- Where do people in your community learn about maternal nutrition?

1. **What do you think maternal nutrition is?**

[probe]:

- What do women eat during pregnancy?
- How many times women eat during pregnancy?
- Are there any preferred foods/drinks for pregnant women?
- Are there any foods/drinks not allowed during pregnancy?

1. **Where women get information about diet/nutrition during pregnancy and the adherence with the advice?**

[Probe]:

- Could you tell me about any advice they have received about what to eat during their pregnancy and who offered or where did they learn about the recommendation?
- Different sources of information: people in the family, relatives outside of the house, health practitioners, radio, TV etc.
- What do you think about the different pieces of advice?
- Would you follow the advice—why and why not?
- Do healthcare professionals (doctors, midwives) provide nutritional information to women?
- What are the types of information provided on maternal nutrition?
- What are the mediums/formats in which information is provided?

1. **How have you been eating during your pregnancy?**

**[Probe]**

- The same as before? More food or less food? Different foods? Why?
- Are you taking any medicines? Vitamins? Tonics? Injections? Home remedies? Herbs?
- Why? With what frequency?

1. **Do you have favourite foods or anything else that you are eating a lot of or that are particularly appealing to you now that you are pregnant?**

[Probe]

- Are you able to get these foods as much as you would like?
- If not, what are hindrances/problems to get these foods?

1. **Now I would like to ask you about your consumption of fruit and vegetables, particularly those that are dark green:**

[Probe]

- Are you eating more, the same or less fruit now than before you were pregnant? Why?
- Which fruit do you prefer?
- Are you eating more, the same or fewer vegetables now than before you were pregnant?
- Why? Which vegetables do you prefer?
- Are these foods good for pregnant women? Which ones and why? If a pregnant woman doesn’t get enough of these foods is there a problem?
- Are any of these foods harmful to a pregnant woman? Which ones and why?

1. **Can you tell me are there any special foods or preparations or products that you are taking as diet supplements while you are pregnant?**

[Probe]:

Vitamin pills?

Tonics?

Herbs?

Foods that are fortified like a cereal product

1. **In this community what do women usually do in terms of their diets? Are there foods that women try to get at different times of their pregnancy or that they try to avoid? Are there foods that are particularly good or bad for the growing foetus?**

**Thank you very much for your answers to my questions. These answers will be very useful indeed to our research project. Is there anything I haven't asked about, that you would like to add?**
